# Naringenin and β-carotene convert human white adipocytes to a beige phenotype and elevate hormone- stimulated lipolysis

**DOI:** 10.3389/fendo.2023.1148954

**Published:** 2023-04-17

**Authors:** Ann A. Coulter, Frank L. Greenway, Dachuan Zhang, Sujoy Ghosh, Cathryn R. Coulter, Sarah L. James, Yanlin He, Luke A. Cusimano, Candida J. Rebello

**Affiliations:** ^1^ Computational Biology, Pennington Biomedical Research Center, Baton Rouge, LA, United States; ^2^ Clinical Trials, Pennington Biomedical Research Center, Baton Rouge, LA, United States; ^3^ Biostatistics, Pennington Biomedical Research Center, Baton Rouge, LA, United States; ^4^ Adjunct Faculty, Pennington Biomedical Research Center, Baton Rouge, LA, United States; ^5^ Brain Glycemic and Metabolism Control, Pennington Biomedical Research Center, Baton Rouge, LA, United States; ^6^ Cusimano Plastic and Reconstructive Surgery, Baton Rouge, LA, United States; ^7^ Nutrition and Chronic Disease, Pennington Biomedical Research Center, Baton Rouge, LA, United States

**Keywords:** UCP1, naringenin, carotenoid, PPARγ, RXRγ, PPARα, adiponectin, lipolysis

## Abstract

**Introduction:**

Naringenin, a peroxisome proliferator-activated receptor (PPAR) activator found in citrus fruits, upregulates markers of thermogenesis and insulin sensitivity in human adipose tissue. Our pharmacokinetics clinical trial demonstrated that naringenin is safe and bioavailable, and our case report showed that naringenin causes weight loss and improves insulin sensitivity. PPARs form heterodimers with retinoic-X-receptors (RXRs) at promoter elements of target genes. Retinoic acid is an RXR ligand metabolized from dietary carotenoids. The carotenoid β-carotene reduces adiposity and insulin resistance in clinical trials. Our goal was to examine if carotenoids strengthen the beneficial effects of naringenin on human adipocyte metabolism.

**Methods:**

Human preadipocytes from donors with obesity were differentiated in culture and treated with 8µM naringenin + 2µM β-carotene (NRBC) for seven days. Candidate genes involved in thermogenesis and glucose metabolism were measured as well as hormone-stimulated lipolysis.

**Results:**

We found that β-carotene acts synergistically with naringenin to boost UCP1 and glucose metabolism genes including GLUT4 and adiponectin, compared to naringenin alone. Protein levels of PPARα, PPARγ and PPARγ-coactivator-1α, key modulators of thermogenesis and insulin sensitivity, were also upregulated after treatment with NRBC. Transcriptome sequencing was conducted and the bioinformatics analyses of the data revealed that NRBC induced enzymes for several non-UCP1 pathways for energy expenditure including triglyceride cycling, creatine kinases, and Peptidase M20 Domain Containing 1 (PM20D1). A comprehensive analysis of changes in receptor expression showed that NRBC upregulated eight receptors that have been linked to lipolysis or thermogenesis including the β1-adrenergic receptor and the parathyroid hormone receptor. NRBC increased levels of triglyceride lipases and agonist-stimulated lipolysis in adipocytes. We observed that expression of RXRγ, an isoform of unknown function, was induced ten-fold after treatment with NRBC. We show that RXRγ is a coactivator bound to the immunoprecipitated PPARγ protein complex from white and beige human adipocytes.

**Discussion:**

There is a need for obesity treatments that can be administered long-term without side effects. NRBC increases the abundance and lipolytic response of multiple receptors for hormones released after exercise and cold exposure. Lipolysis provides the fuel for thermogenesis, and these observations suggest that NRBC has therapeutic potential.

## Introduction

Adipose tissue is a complex, adaptable organ composed of multiple types of adipocytes which vary in function (1). White adipocytes store triglycerides and expand in number and size under conditions of excess energy intake. Brown and beige adipocytes abound in mitochondria and express uncoupling protein 1 (UCP1), a protein that shifts mitochondrial fat oxidation away from ATP production and towards thermogenesis. Increased density of beige adipocytes and UCP1 in fat depots is associated with elevated energy expenditure and resistance to weight gain and type 2 diabetes (2–4). In rodents, white adipose tissues can adapt to a chronic environmental stimulus such as cold exposure by producing beige adipocytes from precursor cells and by converting white adipocytes into beige cells (5). The adaptive response to cold exposure involves release of norepinephrine by sympathetic nerve fibers to activate β3-adrenergic receptors (β3AR) abundantly expressed in adipocytes (6).

In contrast to rodents, human adipocytes express extremely low levels of the βARs. Humans lack a robust response to systemic infusion of β-adrenergic agonists even when combined with cold exposure (7–9). However, synthetic peroxisome proliferator activator receptor (PPAR)α and PPARƴ ligands have been shown to have potent activity in the conversion of primary human white adipocytes to beige UCP1-expressing cells *in vitro* (10, 11). PPARs are ligand-activated nuclear receptors enriched in metabolic tissues, and they regulate UCP1 and many genes controlling fat oxidation and insulin sensitivity by binding to upstream PPAR responsive elements (PPREs). PPARγ is subject to complex cell-specific post-translational regulatory mechanisms and has multiple ligand binding domains (12). Depending on the binding characteristics of a particular ligand, PPARγ can stimulate adipogenesis, insulin sensitivity, fat oxidation or thermogenesis in adipose tissues (13).

Thiazolidinediones (TZDs) are a class of potent PPARγ agonists that have been approved by the United States Food and Drug Administration (FDA) for the treatment of type 2 diabetes. However, TZDs have multiple adverse effects including weight gain, heart failure, and risk for bladder cancer (14, 15). PPARα agonists primarily upregulate genes for lipolysis and mitochondrial β-oxidation of fatty acids in primary human adipocytes (16). A synthetic PPARα activator, fenofibrate, has been approved for treatment of dyslipidemia (17, 18). At this time, there are no FDA-approved PPARγ or PPARα activators for treatment of obesity (19–21).

Despite the complex regulation of ligand binding to PPARγ, evidence is growing that selective modulators and partial agonists can direct activity toward thermogenesis and away from adipogenesis (12, 22, 23). Naringenin (NR), a polyphenol found in citrus fruit, activates transcriptional activity of PPARγ and PPARα in an expression system with a PPRE linked to a reporter gene (24). In a previous study, we treated human subcutaneous adipocytes with NR and saw induction of UCP1 mRNA as well as increases in basal and maximal oxygen consumption rate (OCR) (25). We used selective inhibitors to demonstrate that upregulation of thermogenesis genes by NR requires activation of both PPARγ and PPARα in adipocytes (26). Data from our clinical studies suggest that naringenin is bioavailable and has potential as a treatment for obesity and type 2 diabetes. Our pharmacokinetic clinical trial showed that naringenin is safe and well-tolerated at doses ranging from 150 mg to 900 mg (27). In a case study of an individual with obesity and untreated type 2 diabetes, we found that body weight and fasting insulin concentrations decreased, and there was a measurable increase in energy expenditure after ingestion of NR for eight weeks (26).

Carotenoids are vitamin A precursors found in fruits and vegetables, and their consumption has been shown to reduce fat mass and insulin resistance in children. Baseline blood concentrations of β-carotene (BC) are inversely correlated with fat mass (28, 29). Most carotenoids are metabolized into retinoid ligands for retinoic acid receptors (RAR) and retinoic-X-receptors (RXR), which are transcriptional coactivators for PPARs. The objective of this study was to determine whether carotenoids could amplify the effects of NR on gene expression and function to convert primary human adipocytes to a beige phenotype. We observed synergistic increases in a subset of mRNAs including UCP1, GLUT4, ATGL and adiponectin with the combination of NR and BC (NRBC). Protein levels of PPARα, PPARγ, PGC-1α and NAMPT were selectively upregulated without increases in mRNA levels. Whole transcriptome sequencing was conducted, and the results showed that NRBC induced genes for multiple non-UCP1 energy-dissipating futile cycles, beneficial secreted peptides, and adipokines. Importantly, NRBC increased levels of multiple thermogenesis and lipolysis-linked receptors including the β1- adrenergic receptor (β1AR), parathyroid hormone receptor (PTHR), and the stimulatory ratio of natriuretic peptide receptors (NPR1/NPR3). A comprehensive analysis of lipolysis was conducted, and the results showed that the capacity for PTHR and βAR agonist-stimulated lipolysis was substantially higher in NRBC-treated adipocytes compared to untreated cells. Expression of RXRγ, a unique isoform associated with brown adipogenesis, was upregulated ten-fold after treatment. We observed that RXRγ was bound in the PPARγ transcriptional complex immunoprecipitated from human adipocytes. These results suggest that NRBC has potential as a treatment for obesity and type 2 diabetes and is safe primarily because it acts on peripheral tissues, unlike most obesity medications that act on the central nervous system (CNS) (30).

## Materials and methods

### Chemicals and antibodies

Naringenin extract (NR) from whole citrus sinensis oranges (purity ≥ 30%) was purchased from GE Nutrients, Inc. (Gencor, Irvine, CA). BC, lycopene, and lutein were from Cayman Chemical Co. Protease and phosphatase inhibitors were purchased from Cell Signaling Technology (Danvers, MA), TGX protein gels from BIO-RAD (Hercules, CA). Type 1 collagenase, glycerol standard solution, adenosine, estradiol, human pituitary growth hormone, dobutamine hydrochloride, human atrial natriuretic peptide, ACTH, menthol, 8-CPT-cAMP were purchased from Sigma-Aldrich. Isoproterenol, human parathyroid hormone (1–34), CDCA, were purchased from Cayman Chemicals. Glycerol reagent A was from ZenBio (Durham, NC). All other chemicals were purchased from Sigma (St. Louis, Mo) unless otherwise indicated.

Primary antibodies used were UCP1 (#MAB6158, R&D Systems), GLUT4 (Ab654 Abcam), PGC-1α (ST1202, Sigma) and β-Actin (A5316, Sigma), or monoclonals from Santa Cruz against ATGL (sc-365278), adiponectin (sc-136131), PPARα (398394), PPARγ (sc-7273), NAMPT (sc-393444), RXRγ (sc-514134), RXRα (sc-515929). HRP-linked anti-rabbit (12-348, Sigma), anti-mouse (AP130P, Sigma) and anti-IgG kappa light chain (sc-516102, Santa Cruz) were used to detect specific antibody-antigen complexes. Western Lightning Plus-ECL was from PerkinElmer (Waltham, MA).

### Human adipocyte cell culture

Human adipose-derived stem cells from overweight and obese female donors were purchased from LaCell, LLC (New Orleans, Louisiana) or isolated from lipoaspirate waste donated post-surgery from women with obesity using methods as previously described (31). Cells were seeded, maintained until two days after becoming confluent, and differentiated into adipocytes in the presence of rosiglitazone and isobutylmethylxanthine for five days as previously described (25). Treatments of adipocytes with 8µM NR and 2µM carotenoid, dissolved in DMSO at 1000X, started 5 days after the differentiation period and lasted for seven days in adipocyte maintenance medium with heat inactivated serum, before RNA and protein were isolated from adipocyte cultures.

### RNA extraction and transcriptome sequencing

Total RNA was extracted from cells using Tri-reagent and purified with RNeasy (Qiagen) into nuclease-free water with RNAsecure Reagent (Thermo Fisher Scientific). RNA integrity was assessed using an Agilent Bioanalyzer 2100. RNA samples were diluted to 50 ng/µL and libraries were constructed using Lexogen Quant-Seq 3’ mRNA-Seq Library Prep Kit (SKU015.96) with oligo(dT) priming. Double-stranded cDNA was purified with magnetic beads, libraries were amplified using PCR, and transcripts were indexed, pooled, and forward-sequenced at 50 bp using NextSeq500 (Illumina). BlueBee software was used to analyze alignment and the DESeq2 V1.32.0 package in R V4.1.0, Rstudio V1.4.1717 and biomaRt V2.48.2 were used for differential expression analysis after estimation of possible outlier counts per gene via the Cook’s distance, and their replacement by the trimmed mean over all counts for that gene. Differential gene expression results were computed for 17525 genes of which 3881 genes were differentially regulated at an adjusted p - value¾0.05. Pathway enrichment analysis was carried out via the Gene Set Enrichment Analysis (GSEA) tool (32) by estimating enrichment on pathways present in the Kyoto Encyclopedia of Genes and Genomes (KEGG) database (33) available from the Molecular Signatures Database repository (MSigDb, http://software.broadinstitute.org/gsea/msigdb) (34), and additional custom pathways. Gene-sets with FDR ≤ 5% were considered as significantly enriched (35).

### Quantitative real time polymerase chain reaction

Quantitative reverse transcriptase and real-time PCR were conducted in one reaction with the gene-specific reverse PCR primer also priming the cDNA synthesis as previously described (25). Primer-probe sequences for UCP1, GLUT4, ATGL, adiponectin, PGC-1α and ribosomal RPL13A, used to adjust target gene values for total RNA in each sample, have been previously reported (25). Additional primer and probe oligonucleotide sets used in this study are shown in 5’ to 3’ orientation: PPARα Forward GTCGATTTCACAAGTGCCTTTC reverse CAGGTAAGAATTTCTGCTTTCAGTT probe AACGAATCGCGTTGTGTGACATCC; PDK4 forward CTGAGAATTATTGACCGCCTCT reverse GAAATTGGCAAGCCGTAACC probe TACATACTCCACTGCACCAACGCC; PPARγ forward CCCAAGTTTGAGTTTGCTGTG reverse GCGGTCTCCACTGAGAATAATG probe TGGAATTAGATGACAGCGACTTGGCA; NAMPT forward TGTTCCTTCAAGTGTAGCTATGT reverse TGCTGGCGTCCTATGTAAAG probe AACGTCTTCAAGGACCCAGTTGCT; CKMT1 forward CTTGACCTGTCCATCTAACCTG reverse ACTCCTCCAGTACCACGTT probe AGATAGCCGCTTCCCAAAGATCCTG. Predesigned human primer and probe sets from Thermofisher Scientific are PM20D1 Hs00399438_m1; ANGPTL4 Hs00211522_m1; GDF11 Hs00195156_m1; S100B Hs00902901_m1.

### Protein extraction, PPARγ immunoprecipitation and Western blotting

Whole cell protein was isolated from differentiated cell cultures after treatment for seven days, lysed with RIPA buffer containing protease and phosphatase inhibitors, and pushed through a 20-gauge needle four times to disrupt organelles. Fifty micrograms of total cell protein were loaded per lane and resolved in 7.5% SDS-PAGE gels, transferred to nitrocellulose membranes, and probed overnight at 4°C with specific primary antibodies. Anti-rabbit or -mouse secondary antibodies conjugated to horseradish peroxidase were used for detection of target proteins. Image J was used to quantify protein bands on Western blots. To adjust for variations in total protein loaded in each lane, β-actin was used.

PPARγ was immunoprecipitated from whole cell lysates with SC-7273 antibody (Santa Cruz) using the Pierce MS-compatible magnetic IP kit with protein A/G beads according to kit directions (Pierce 90409, Thermofisher). Briefly, primary anti-PPARу antibody was incubated with 600 micrograms of adipocyte protein lysate for four hours at 4°C on a tube inverter. The antibody-lysate mixture was then incubated with magnetic protein A/G beads for one hour, and complexes of PPARγ protein attached to beads were isolated, washed and eluted using a magnetic tube holder. Samples were evaporated to dryness under vacuum, resuspended in RIPA buffer and subjected to Western Blot analysis. An HRP-conjugated anti- IgG kappa light chain secondary antibody was used to eliminate primary antibody heavy chain bands from interfering with detection of immunoprecipitated target proteins. Proteins were visualized by chemiluminescence using Western Lightening (Amersham).

### Lipolysis assay

Cells were differentiated in 96-well plates and treated with cell medium (untreated) or NRBC for seven days. On the day of the lipolysis assay, cells were exposed for four hours to buffer (KRB with 1% BSA) or receptor agonists dissolved in buffer. Agonists were used at concentrations shown to give maximum responses and were: 8-Cpt-cAMP 200µM non-hydrolyzable PKA activator of maximum stimulated lipolysis (cAMP) (36), atrial natriuretic peptide 0.1 µM for NPR1 and NPR3 (37), parathyroid hormone (amino acids 1-34) 1 µM for PTHR (38), isoproterenol 1µM all βARs and dobutamine 1µM for β1AR (39), estradiol 1µM for GPER (40), growth hormone 250ng/ml for GHR (41), adrenocorticotropin hormone 1µM for MC1R (42), bile acid chenodeoxycholic acid 30µM for TGR5 (43), adenosine 1µM for ADORA1 and ADORA2B (44), and menthol 100µM for TRPM8 (45). Supernatants were removed for measurement of glycerol released using Glycerol Reagent A, and concentrations were determined using a standard curve. Data are from four or five experiments each with cells from different donors with BMI from 27 to 36.

### Statistical analysis

All statistical analyses were performed using SAS 9.4 (SAS Institute, Cary, North Carolina). The synergistic effect of the combination of NR and BC was investigated by implementing linear mixed effect models including plate as the random effect. The goal was to test if the combination (NRBC) induced a greater response compared to the sum of the individual components’ effects. Based on the mixed effect model, an F test was constructed to evaluate the null hypothesis that the additive effect of NR over control and BC over control was no different from that of NRBC over control. We also used linear mixed effect models to test the effects of: 1) NR, BC, and NRBC compared to control for the target variables of mRNA and protein; 2) Agonist stimulated lipolysis in NRBC pre-treated cells v. untreated cells. All experiments were repeated at least three times in primary adipocytes from different donors. Significance was set at p < 0.05. Data are reported as least squares means ± standard error unless otherwise specified.

## Results

### Carotenoids act synergistically with NR to amplify UCP1 levels in adipocytes

**Figure 1:**
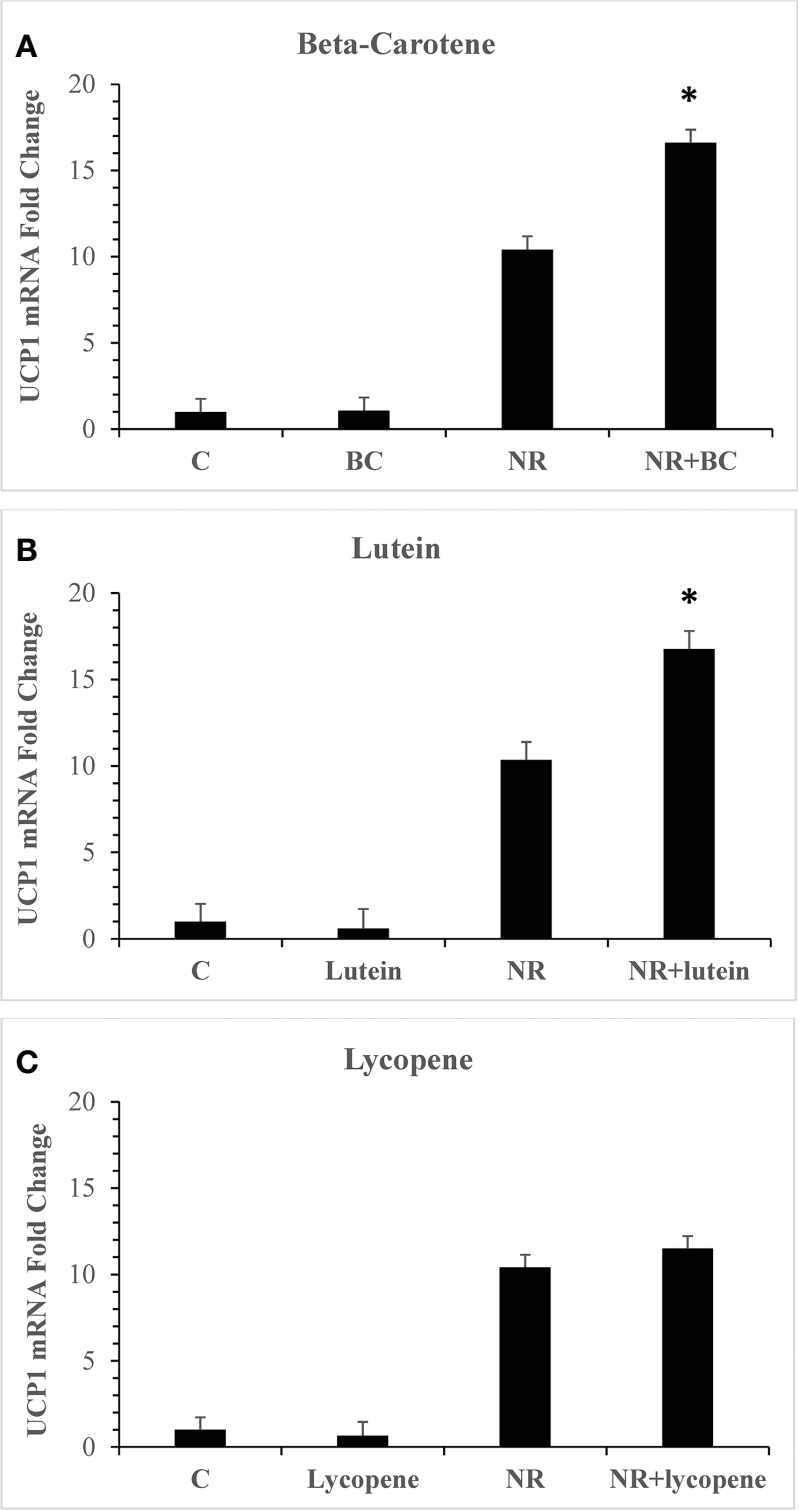
Pro-vitamin A carotenoids and NR act synergistically to elevate levels of UCP1 mRNAs Adipocytes from 4 donors with obesity were treated for seven days with vehicle (control) or 8µM NR and 2µM carotenoids. **(A)** NR+BC **(B)** NR+Lutein **(C)** NR+Lycopene mRNA levels were measured using quantitative RTPCR. Data is expressed in least squares means ± standard error. Synergy for mRNA was calculated as: sum of differences ((NR- Control) + (BC - Control)) *vs* (NRBC - Control). *p < 0.001 for synergy, sum of differences versus NRBC. NR, naringenin; BC, beta carotene.

Metabolites of pro-vitamin A carotenoids are ligands of the retinoid X receptor (RXR) family of nuclear receptors which can heterodimerize with PPARα and PPARγ and recruit coactivators into active transcriptional complexes (46). Our first objective was to evaluate whether treatment of adipocytes with carotenoids could elevate the levels of UCP1, a PPAR target gene known to be upregulated by NR and a marker for beige adipogenesis (25). Three of the most abundant carotenoids in foods and in human serum, BC, lycopene, and lutein were tested (47). Steady state plasma concentrations are approximately 2μM in individuals who eat diets rich in carotenoids, and we used this concentration in experiments (48). Since the mean serum concentration of NR after ingestion of 150mg is 8µM (27), we used this concentration in all assays. Treatment of primary human adipocyte cultures with BC, lutein or lycopene individually for seven days had no effect on gene expression (**Figure 1**). When BC or lutein were combined with NR for treatment, the increase in UCP1 gene expression was synergistic, greater than the sum of the individual effects of each compound (P<0.001). In contrast, lycopene did not alter NR-stimulated UCP1 levels. Unlike lycopene, BC and lutein are pro-vitamin A carotenoids that are metabolized into retinoic acid (49). These data are consistent with a hypothesis that RXR ligands act synergistically with NR to boost thermogenesis gene expression.

### BC elevates the effects of NR on a subset of genes for thermogenesis and insulin sensitivity

**Figure 2:**
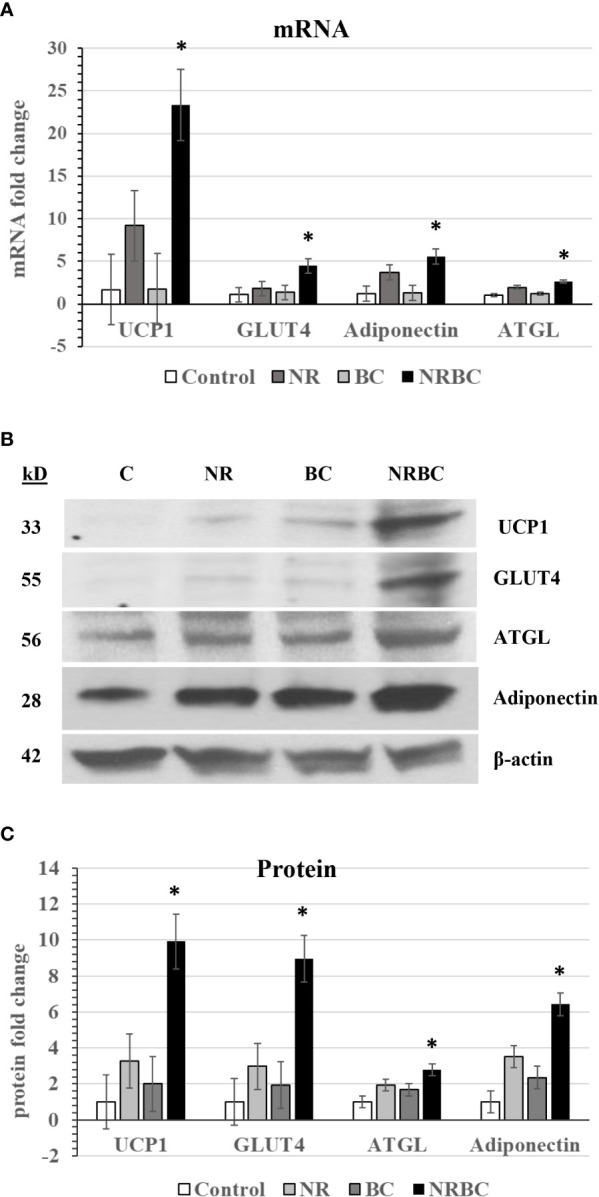
NR and BC synergistically induce metabolism genes **(A)** mRNA levels **(B)** Western Blots of Protein levels **(C)** Protein levels were measured with β-actin as loading control. Adipocytes from three or more donors with obesity were treated for seven days. mRNA data are expressed as least squares means ± standard error. Synergy for mRNA was calculated as: sum of differences ((NR- Control) + (BC - Control)) *vs* (NRBC - Control). *p < 0.05 for synergy. NR, Naringenin; BC, beta carotene; NRBC, naringenin and beta carotene.

We next evaluated the effects of NR, BC and NRBC on expression of additional genes. BC was chosen for the rest of the study because it is safe, stable, bioavailable and has a long half-life in circulation (50). Adipocytes from five donors with body mass index (BMI) ranging from 27 to 36kg/m^2^ were treated with NRBC for seven days. NRBC synergistically boosted mRNA levels for UCP1 (p<0.005), GLUT4 (p<0.02), ATGL (p<0.04) and adiponectin (p<0.05) compared to NR or BC alone (**Figure 2A**). Protein levels showed a similar trend (**Figures 2B, C**). Uncropped Western blots are shown in **Supplemental Figure 1**. ATGL is the rate-limiting lipase for hydrolysis of fatty acids from triglycerides (TGs) (51). GLUT4 is a transporter for glucose uptake, and its upregulation in adipocytes stimulates a cascade of events that reduce insulin resistance (52, 53). Adiponectin is a key circulating factor that acts on muscle and other tissues and improves whole body glucose homeostasis (54). In a previous study we reported induction of CPT1β mRNA in adipocytes after NR treatment (25). We did not see an increase in CPT1β levels with NRBC treatment in comparison to levels induced by NR alone.

**Figure 3:**
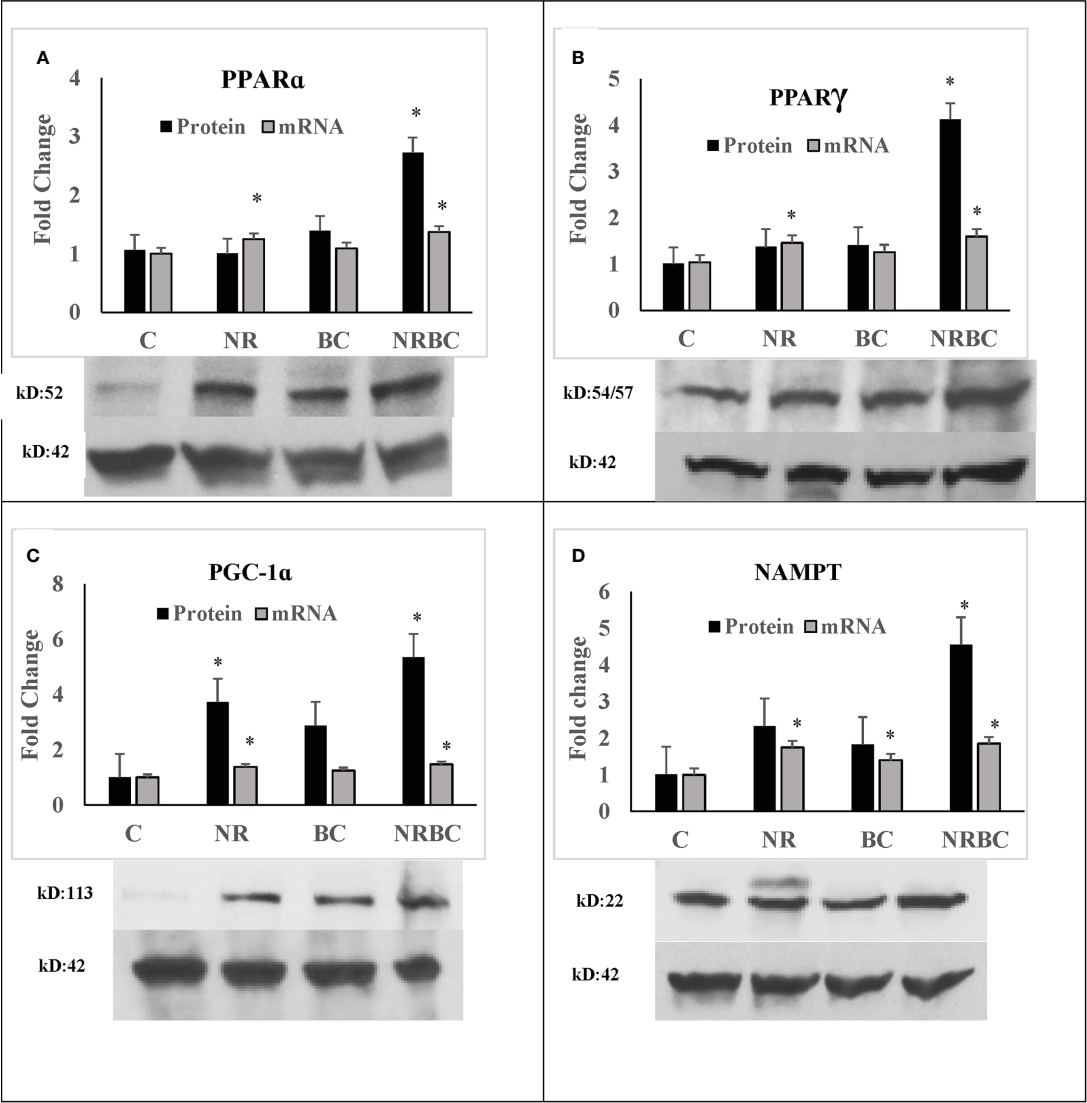
NRBC upregulates a subset of key regulatory proteins without mRNA increases **(A)** PPARα; **(B)** PPARγ; **(C)** PGC-1α; and **(D)** NAMPT. Adipocytes from three to five donors with obesity were treated for seven days. Protein was measured by Western Blotting with β-actin used to adjust for loading. mRNA was quantified by real-time PCR. Data are expressed as least squares means ± standard error, *p < 0.001 NR, Naringenin; BC, beta carotene; NRBC, naringenin and beta carotene.

NRBC upregulated protein levels of PPARα, PPARγ, PGC-1α, and NAMPT three to six-fold (**Figure 3**, p<0.001) without comparable increases in mRNA levels. Uncropped Western blots are shown in **Supplemental Figure 2**. PPARα, PPARγ, and PGC-1α proteins have a short half-life and are rapidly degraded by ubiquitin-proteosome systems (55–57). Inhibition of degradation increases protein levels and target gene expression (58). The binding of ligands and coactivators can influence the turnover rates of PPARα and PPARγ (59, 60). Treatment with NR alone did not upregulate protein levels, indicating that RXR ligand is required in addition to PPAR ligand for protein upregulation. The increase in these proteins in the absence of concurrent upregulation of their transcripts suggests that NR and BC act together to stabilize protein levels through post-translational mechanisms.

### Whole transcriptome sequencing for comprehensive analysis of metabolic effects

**Figure 4:**
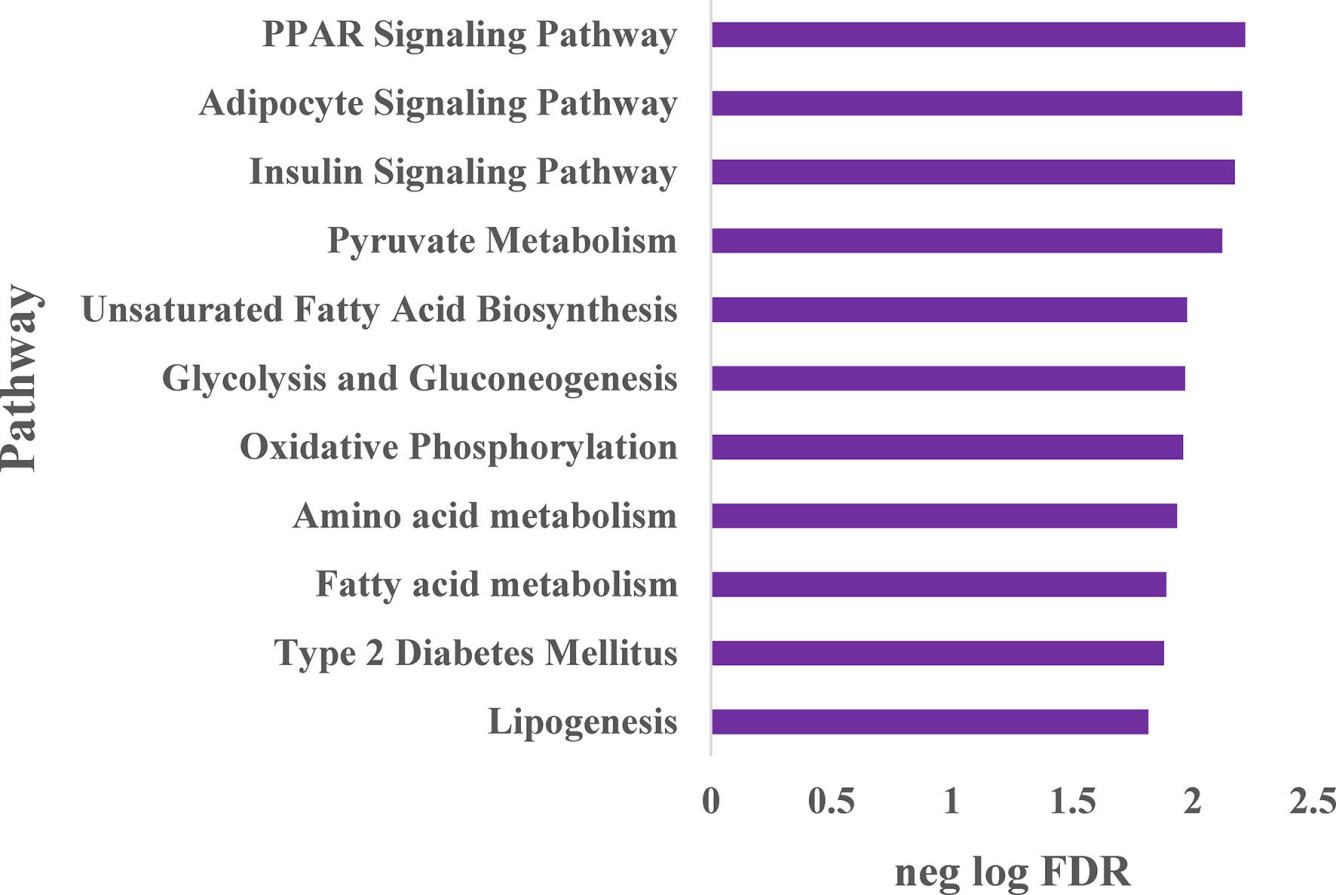
Whole transcriptome sequencing analysis of pathways stimulated by naringenin and β-carotene (NRBC) Adipocytes from two donors with BMI of 27 and 36 kg/m^2^ were treated with cell medium (vehicle control) or NRBC for seven days. cDNA libraries from expressed transcripts were constructed, sequenced and differential gene expression was analyzed. Gene-sets with false discovery rate (FDR) ≤ 5% were considered as significantly enriched, and the top pathways are shown.

**Figure 5:**
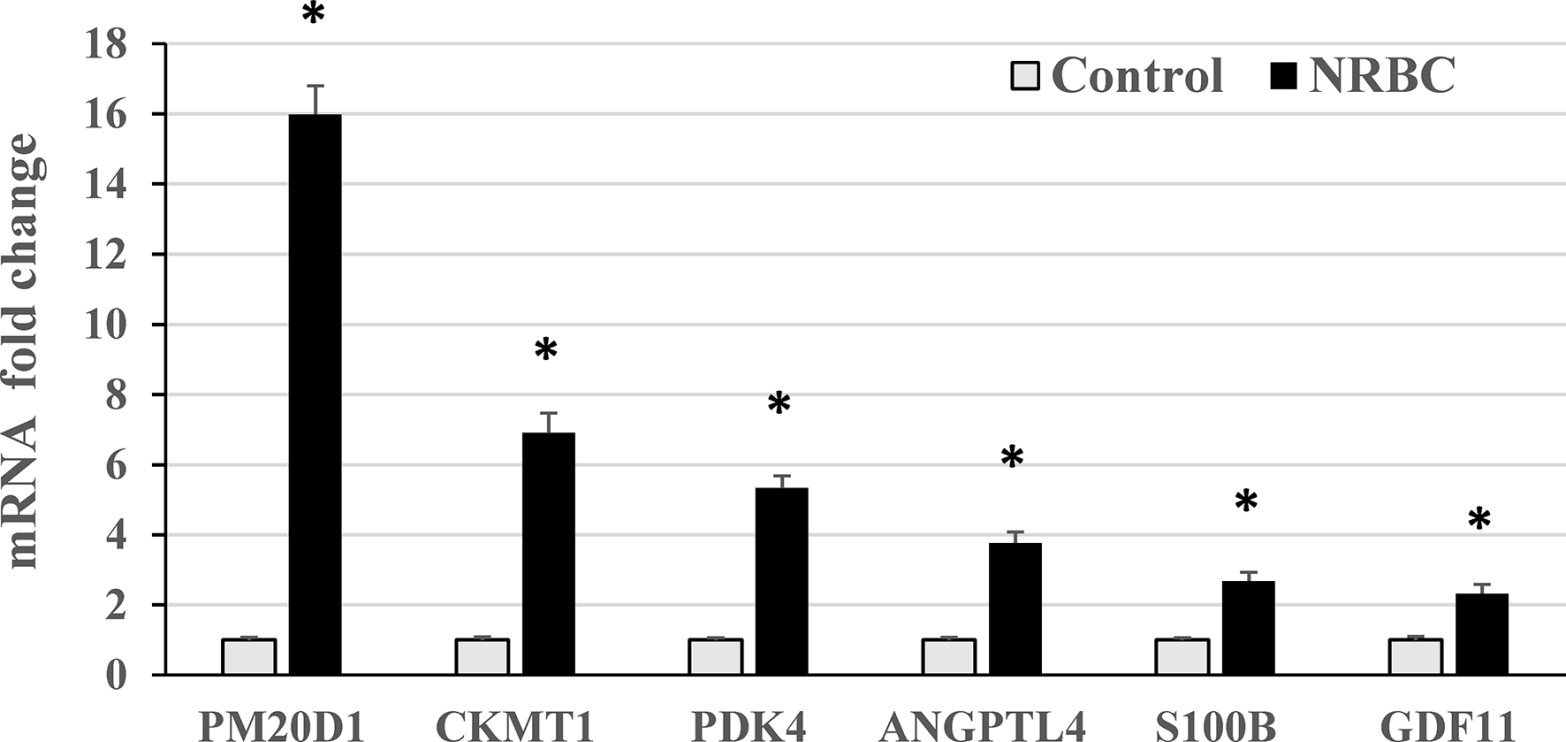
RT-PCR validation of genes upregulated by naringenin and beta carotene (NRBC) in RNA sequencing analysis. Adipocytes from three donors with obesity were treated for seven days. mRNA levels were measured using quantitative RT-PCR. Data are expressed as mean ± standard error, *p < 0.001.

To expand our understanding of human adipocyte reprogramming by NRBC, we conducted whole transcriptome sequencing. Adipocyte cultures from two female donors with overweight and obesity were treated with cell medium (vehicle control) or NRBC for seven days and RNA samples were processed for library construction. Differential gene expression results were computed for 17525 genes of which 3881 genes were significantly regulated at an adjusted p-value <0.05. Pathway enrichment analysis identified PPAR signaling, adipocyte signaling and insulin signaling pathways to be the most significantly increased after treatment (**Figure 4**). In addition, the data analysis showed increases in genes for metabolism of pyruvate, fatty acids, glucose, and amino acids. These changes in mRNA levels were validated for selected genes by qRT-PCR (**Figure 5**, p<0001).

#### PPARγ and PPARα target genes

NRBC robustly upregulated a number of classical brown and beige genes that have previously been identified as targets of synthetic PPARα and PPARу ligands (10) including CIDEA, CITED, perilipins 2, -4, -5, PDK4, GK, AQP7, fatty acid elongases ELOVL 3, -5, -6, ACSL5 and the fatty acid binding proteins FABP3, FABP4 and FABP7 (**Table 1**). In addition, FABP5, which delivers retinoic acid to nuclear receptors was significantly upregulated.

**Table 1:** NRBC-induced genes associated with brown/beige phenotype previously shown as upregulated by synthetic PPARγ and PPARα activators.

Gene	Description	Fold	Padj*
CIDEA	cell death inducing DFFA like effector a	12.4	4.04E-06
CITED1	Cbp/p300 interacting transactivator domain 1	4.3	1.37E-11
PLIN2	perilipin 2	1.8	2.56E-13
PLIN4	perilipin 4	4.1	4.16E-49
PLIN5	perilipin 5	3.0	1.39E-11
AQP7	aquaporin 7 glycerol channel	3.5	4.26E-20
ELOVL3	ELOVL fatty acid elongase 3	2.4	5.38E-07
ELOVL5	ELOVL fatty acid elongase 5	2.2	2.46E-26
ELOVL6	ELOVL fatty acid elongase 6	2.3	5.71E-13
ACSL5	acyl-CoA synthetase long chain family member 5	1.8	0.017
FABP3	fatty acid binding protein 3	2.5	2.65E-14
FABP4	fatty acid binding protein 4	5.6	2.42E-89
FABP7	fatty acid binding protein 7	6.0	2.32E-09
FABP5	fatty acid binding protein 5	1.9	1.1E-05

*Adjusted p-value.

#### Upregulation of secreted peptides and adipokines

Adipose tissue is an endocrine organ that secretes proteins, hormones, and bioactive lipids with beneficial paracrine effects on whole-body fat and glucose metabolism. NRBC treatment significantly upregulated the expression of a number of these genes including ANGPTL4, FNDC4 and GDF11 (**Table 2**). ANGPTL4 is produced by adipocytes and promotes lipolysis (**Table 2**) (61). Both the full-length protein and a truncated form of ANGPTL4 are secreted, and their overexpression in mice stimulates energy expenditure, lowers adiposity, and converts white fat to the beige phenotype (62). FNDC4 induces UCP1 and beige genes and promotes insulin sensitivity in adipocytes (63, 64). GDF11, a circulating cytokine in the transforming growth factor β superfamily, declines with age and has been under investigation as an anti- aging therapeutic (65). Restoration of circulating levels in aged mice promotes adiponectin secretion by fat tissues and reduces adiposity (66).

**Table 2:** Genes encoding secreted lipokines and peptides upregulated by NRBC.

Gene	Description	Fold	Padj*
ANGPTL4	angiopoietin like 4	3.1	1.4E-34
FNDC4	fibronectin type III domain containing 4	1.7	3.6E-08
GDF11	growth differentiation factor 11	2.2	2.6E-24
CYP4F11	cytochrome P450 family 4 subfamily F member 11	14.0	3.0E-05
EPHX1	epoxide hydrolase 1	1.7	3.2E-06
EPHX2	epoxide hydrolase 2	1.6	6.2E-04
NMB	neuromedin B	2.7	1.9E-30
POMC	proopiomelanocortin	1.7	6.0E-06
PCSK1	proprotein convertase subtilisin/kexin type 1	1.7	4.4E-03
S100B	S100 calcium binding protein B	2.5	5.0E-11

*Adjusted p-value.

Hydroxyeicosatetraenoic acids (HETEs) and dihydroxyoctadecanoic acids (diHOMEs) are secreted bioactive lipids that are produced from cytochrome P450 metabolites in brown adipocytes after cold exposure and exercise (67, 68). CYP4F11, a cytochrome that produces 20-HETE from arachidonic acid (69), was abundantly increased by NRBC. Circulating levels of 20-HETE correlate with elevated energy expenditure after cold exposure in people with detectable levels of BAT (70), and it is a PPARα activator (71). In addition, NRBC induced epoxide hydrolases EPHX1 and EPHX2, enzymes that utilize HETEs to produce 12,13-diHOME, a lipokine associated with improved insulin sensitivity and reduced triglycerides after exercise (72, 73).

Several circulating CNS-acting proteins were increased by NRBC. Neuromedin B is a peptide that acts on hypothalamic neurons to promote satiety, and a missense mutation is linked to hyperphagia and obesity in genetic studies (74, 75). Pro-opiomelanocortin (POMC), a peptide prohormone that is cleaved by PCSK1 into the hormone α-MSH, is involved in the suppression of food intake (76). POMC and PCSK1 were both elevated by NRBC in adipocytes. The important role of POMC is shown in studies linking severe obesity in humans to rare genetic mutations in POMC or PCSK1 (77). S100b, which has been characterized in brown adipocytes of mice, stimulates neurite outgrowth of sympathetic terminals in adipose tissue following cold exposure (78). The upregulation of genes for CNS-acting circulating factors suggests that appetite reduction could play a role in the physiological response to NRBC.

#### Increases in thermogenesis genes for non-UCP1 uncoupling and substrate cycling pathways

Several mechanisms other than uncoupling of mitochondria by UCP1 can yield thermogenic energy expenditure. These mechanisms were originally identified in studies of brown adipose tissue (BAT) from cold exposed Ucp1-knockout mice that are able to maintain their body temperature (79). We found that NRBC induced significant increases in a number of novel thermogenesis genes in human adipocytes (**Table 3**). RXRγ, a retinoic acid receptor isoform of unknown function, was highly upregulated. This isoform is localized in UCP1 positive cells in human adipose tissues and is expressed at elevated basal levels in adipose-derived stem cells that subsequently differentiate into UCP1-expressing brown adipocytes (80). These observations suggest that RXRγ may be a key transcription factor in the differentiation of human brown and beige adipocytes.

**Table 3:** Thermogenesis genes upregulated by NRBC.

Gene	Description	Fold	Padj*
RXRG	retinoid X receptor gamma	10.0	8.2E-19
PM20D1	peptidase M20 domain containing 1	15.7	2.2E-07
CKMT1A	creatine kinase, mitochondrial 1A	5.1	3.7E-05
CKMT1B	creatine kinase, mitochondrial 1B	3.9	3.5E-06
CKMT2	creatine kinase, mitochondrial 2	3.8	5.1E-18
PDK4	pyruvate dehydrogenase kinase 4	4.2	1.0E-80
GPD1	glycerol-3-phosphate dehydrogenase 1	3.3	1.8E-25
GK	glycerol kinase	2.4	5.4E-03
PCK1	phosphoenolpyruvate carboxykinase 1	3.8	6.0E-08
AIFM2	apoptosis inducing factor mitochondria associated 2	2.0	1.7E-29
UCP2	uncoupling protein 2	3.4	2.5E-31
NAMPT	nicotinamide phosphoribosyltransferase	2.1	3.4E-23
NAPRT	nicotinate phosphoribosyltransferase	2.4	3.1E-22
LIPE	hormone sensitive lipase	3.2	2.5E-31
PRKAR2B	Protein kinase cAMP-dep type II regulatory subunit B	2.5	3.5E-20

*Adjusted p-value.

PM20D1 levels were upregulated approximately 15-fold in adipocytes treated with NRBC, and human genetic studies have shown that the PM20D1 gene promoter has a PPRE (81). PM20D1 is a secreted enzyme that regulates synthesis and degradation of N-acyl amino acids (NAAs), molecules that directly uncouple mitochondria and increase energy expenditure (82). High PM20D1 levels in the white adipose tissue of mice correlates with increased respiration, reversal of high fat diet-induced obesity, and reductions in blood glucose (82–84). Creatine phosphate cycling is an enzymatic pathway that contributes to thermogenesis in Ucp1-knockout mice (85). The synthesis and breakdown of creatine phosphate by the mitochondrial creatine kinases CKMT1A, CKMT1B and CKMT2 releases heat and consumes ATP. We found strong upregulation of the genes for all three CKMT isozymes after NRBC treatment of white adipocytes. In humans, CKMT proteins have previously only been detected in primary brown adipocytes (86).

Beige cells favor utilization of fatty acids rather than glucose to fuel thermogenesis and have high lipase activity (87). In addition to ATGL, hormone sensitive lipase (HSL) and protein kinase A regulatory subunit 2A (PRKAR2B) were upregulated by NRBC treatment (**Table 3**). PRKAR2B is the key PKA subunit regulating activation of lipolysis subsequent to ligand binding of Gs-coupled receptors (88). NRBC significantly upregulated genes for futile cycling of triglycerides, PDK4, GPD1, GK and PCK1. The activities of these enzymes result in shuttling of glycolytic intermediates into pyruvate for the synthesis of glycerol and glycerol-3-phosphate, the precursors for re-esterification of fatty acids during TG synthesis (10). NRBC also stimulated increases in AIFM2, an NADH oxidase (AIFM2) that supports glucose metabolism during thermogenesis in brown adipocytes (89), and UCP2. Elevated UCP2 levels are associated with cells that have high fatty acid oxidation rates, and evidence suggests that UCP2 facilitates fatty acid oxidation to fuel mitochondrial thermogenesis (90–92).

Nicotinamide phosphoribosyltransferase (NAMPT) and nicotinic acid phosphoribosyltransferase (NAPRT) encode enzymes that produce nicotine adenine dinucleotide (NAD) and are upregulated over two-fold. NAD is a key cofactor for cellular metabolism enzymes. Impaired NAD synthesis in adipocytes causes systemic insulin resistance and suppresses lipolysis and thermogenesis in mice (93, 94). In human adipocytes, increasing intracellular NAD induces UCP1 and mitochondrial biogenesis, which are markers of beige cells (95). In summary, these changes in thermogenesis genes suggest that NRBC-treated adipocytes have a higher capacity for uncoupled respiration and thermogenic futile cycling pathways.

#### NRBC increases expression of receptors that regulate lipolysis and thermogenesis

**Figure 6:**
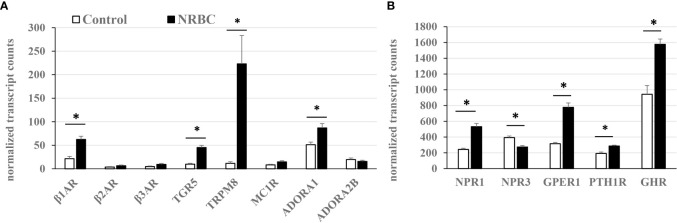
Relative receptor levels in white adipocytes (untreated) and NRBC-treated cells. **(A)** Low abundance **(B)** High abundance. Adipocytes from two donors with obesity were treated with cell medium (Control) or NRBC for seven days and transcript sequencing analysis was conducted. Data are expressed as mean normalized transcript counts (DEseq2). * indicates Padj< 0.002 for Control *vs* NRBC. β-adrenergic receptors (β1AR, β2AR, β3AR), G-protein coupled bile acid receptor (TGR5), Transient receptor potential cation channel subfamily M member 8 (TRPM8), Melanocortin-1 receptor (MC1R), Adenosine receptors A1 and A2B (ADORA1, ADORA2B), Natriuretic peptide receptors (NPR1, NPR3), G-protein coupled estrogen receptor 1 (GPER1), Parathyroid hormone receptor 1 (PTHR1), Growth hormone receptor (GHR).

There is little comprehensive data available on relative receptor levels or hormone-stimulated lipolysis in human adipocytes. Based on our RNA sequencing data, receptor abundance was estimated from transcript reads per total number of kilobases sequenced (**Figures 6A, B**). NRBC treatment significantly increased expression of eight receptors potentially capable of driving lipolysis or thermogenesis through various mechanisms including the β1AR, bile acid receptor TGR5, cold receptor TRPM8, adenosine receptor ADORA1, NPR1, G-protein coupled estrogen receptor-1 (GPER1), growth hormone receptor (GHR) and PTHR1 (Padj<0.002). In addition, there were increases in β2AR, β3AR, and melanocortin-1 receptor (MC1R) that did not achieve statistical significance due to variability in the response. The β2AR and β3AR had the lowest expression levels compared to all other receptors in white adipocytes. The β1AR was approximately four times more abundant than the β2AR and β3AR and increased another three-fold after NRBC exposure (**Figure 6A**). TGR5, TRPM8 and ADORA1 and ADORA2 were also expressed at low levels in untreated white adipocytes.

The ratio of stimulatory NPR1 to the clearance receptor NPR3 determines the magnitude of the response to natriuretic peptides. NRBC treatment increased the NPR1/NPR3 ratio four-fold in NRBC- treated adipocytes, suggesting that adipocytes would be more responsive to natriuretic peptides (**Figure 6B**). PTHR levels were upregulated over fifty percent by NRBC (**Figure 6B**). GHR was the most abundant of all receptors upregulated by NRBC, and studies suggest that GH triggers lipolysis by unique mechanisms that occur downstream of receptor signaling in human adipocytes (41, 96). GPER1 is abundantly expressed in adipocytes and estradiol is the endogenous ligand. Little is known about the role of GPER1 in white adipocytes (97). A selective synthetic agonist for GPER stimulates weight loss and energy expenditure in mice (98).

### NRBC-treated adipocytes have higher agonist-stimulated lipolysis than white adipocytes

Thermogenesis is fueled by lipolysis, so we determined whether agonist-stimulated glycerol release, a measure of lipolysis, reflected the increases in receptors and lipolysis machinery in NRBC-treated adipocytes. First, adipocyte cultures from four donors who had obesity were treated with either cell medium (untreated control cells) or NRBC for seven days. On the day of the acute lipolysis assay, cells were exposed to the individual receptor agonists for four hours in buffer and glycerol released into the cell supernatant was measured. Non-hydrolyzable 8-cpt-cAMP, a potent activator of PKA signaling, was also evaluated in each experiment as a positive control since it bypasses individual receptors and measures the maximum capacity for lipolysis (36). The βARs, PTHR, MC1R, GPER and ADORA2B are all stimulatory G-protein (Gs) coupled receptors and signal through the cAMP-PKA signal transduction mechanism. NPR1 is not a Gs-coupled receptor and signals through an alternate mechanism of guanylate cyclase-cGMP-protein kinase G (PKG).

**Figure 7:**
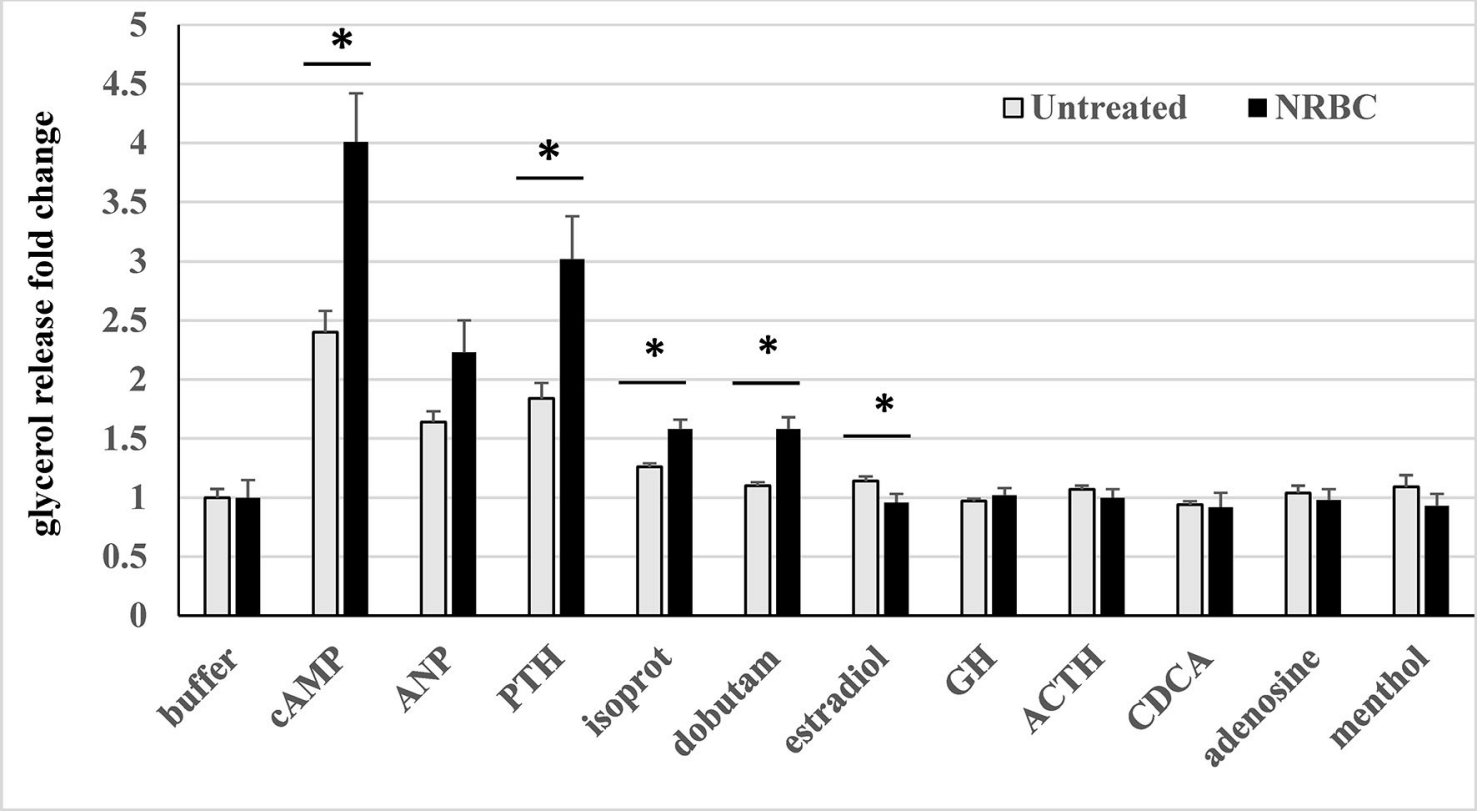
Hormone-stimulated lipolysis in white adipocytes (untreated) and NRBC-pretreated cells. After 7d pretreatment with vehicle (untreated) or NRBC, adipocytes were exposed for 4 hours to receptor agonists in KRB buffer. Supernatants were removed for measurement of glycerol. Data are presented as least squares mean ± standard error from experiments using cells from four different donors with BMIs ranging from 27 to 36, each with at least 6 replicates. (*indicates a difference between untreated white adipocytes and NRBC-treated adipocytes p≤ 0.02) cAMP 8-Cpt-cAMP 200µM, ANP atrial natriuretic peptide 0.1 µM, PTH parathyroid hormone (1-34) 1 µM, isoprot isoproterenol 1µM, dobutam dobutamine 1µM, estradiol 1µM, GH growth hormone 250ng/ml, ACTH adrenocorticotropin hormone 1µM, CDCA chenodeoxycholic acid 30µM, adenosine 1µM, menthol 100µM.

Of all the hormones tested, PTH- stimulated lipolysis was the highest in control white adipocytes and increased by the greatest magnitude in cultures exposed to NRBC (**Figure 7**). PTH treatment of human adipocytes was previously shown to stimulate UCP1 expression and oxygen consumption (99). We tested atrial natriuretic peptide (ANP), one of the key endogenous ligands for NPR1 released from atrial myocytes in response to increases in blood pressure and environmental stimuli such as exercise and cold exposure (100, 101). ANP-stimulated lipolysis increased in NRBC-treated cells, but the overall effect was not statistically significant in adipocytes from four donors.

The collective activity of the βARs was measured using isoproterenol, a non-selective agonist of all three βARs, to evaluate the overall potential for increased sensitivity to sympathetic stimulation. Since the β1AR was 5-fold more abundant than the other βARs, the β1AR-selective ligand dobutamine was also tested. Isoproterenol- and dobutamine- stimulated lipolysis were significantly boosted in NRBC-treated cells, and dobutamine activity was comparable with isoproterenol activity (**Figure 7**). In NRBC-treated adipocytes, lipolysis stimulated by 8-cpt-cAMP was significantly higher than levels in control cells, showing that maximum capacity for lipolysis was elevated in parallel with the upregulation of the PKA regulatory subunit and lipases.

Agonists for the receptors GHR, MC1R, ADORA1/ADORA2B, TGR5 and TRPM8 did not stimulate detectable increases in lipolysis. In murine white and brown adipocytes adrenocorticotropic hormone (ACTH) and adenosine, endogenous ligands for melanocortin receptors and ADORA2B respectively, stimulate lipolysis and OCR (42, 44, 102). Bile acids activate TGR5 in human brown adipocytes and increase OCR (43). Although GPER activation by estrogen stimulates cAMP production in cancer cells (36), our data showed a small reduction in lipolysis.

### RXRγ is bound to PPARγ complexes before and after treatment

**Figure 8:**
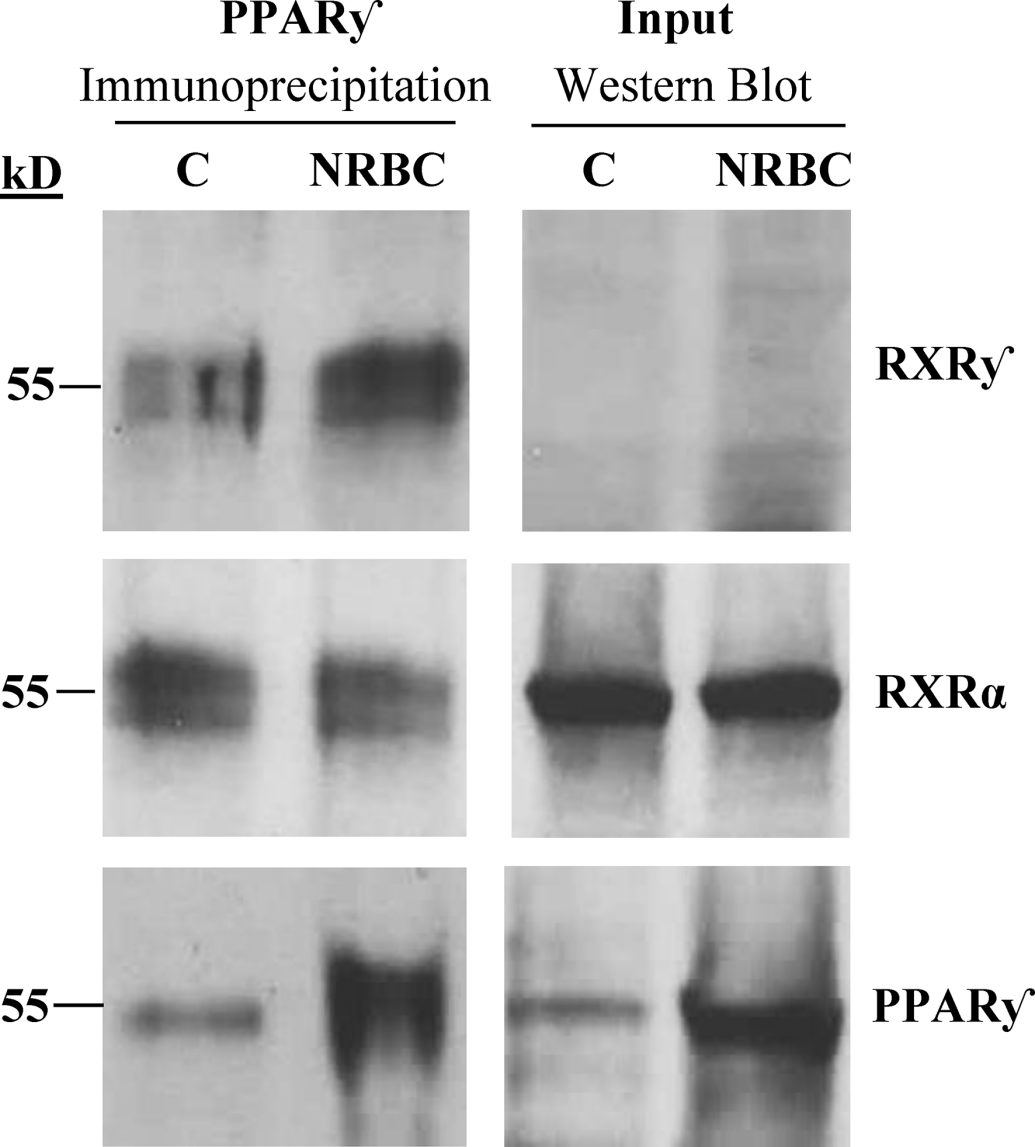
Analysis of RXR isoforms bound to immunoprecipitated PPARγ complexes. Adipocytes were treated with vehicle or NRBC for seven days. RXRγ, RXRα and PPARγ protein levels were analyzed by Western blotting. For analysis of whole cell protein levels (input), 50 μg of protein lysate was loaded in each lane. PPARγ was immunoprecipitated from 600 μg of the same whole cell lysate for analysis of PPARγ cofactor binding. An anti-IgGκ light chain secondary antibody was used for detection. This experiment was repeated three times with adipocytes from obese donors.

NRBC induced a strong increase in RXRγ mRNA after treatment and RXRα was unchanged. RXRγ is a unique isoform associated with differentiation of brown adipocytes in human adipose tissue (80). RXRs can form homodimers or heterodimers with multiple nuclear receptors other than PPARγ, so we evaluated whether RXRγ was bound to PPARγ after NRBC treatment. PPARγ was immunoprecipitated from protein lysates of untreated and NRBC- treated adipocytes. The protein complexes pulled down with PPARγ were analyzed on Western Blots (**Figure 8**). RXRγ protein expression was too low for detection on regular Western Blots of whole cell protein, consistent with our transcriptome sequencing data indicating that it has a low copy number. However, we observed that RXRγ was visible in the immunoprecipitated PPARγ protein complex in control cells (white adipocytes) and in NRBC treated beige adipocytes (**Figure 8** **).** RXRα is more abundantly expressed and was readily detected in Western Blots and in PPARγ immunoprecipitates. These results suggest that both isoforms are bound constitutively and that binding of RXRу is independent of the addition of exogenous ligands. RXR isoforms stabilize PPARγ complexes at PPRE promoter elements and direct expression of specific target genes subsequent to ligand binding (46). Therefore, elevated levels of RXRγ may direct expression towards beige genes after the addition of BC.

## Discussion

Thermogenesis is a fundamental component of energy balance. A number of studies have demonstrated that human adipocytes have the functional plasticity to be transformed into thermogenic cells by non-adrenergic stimuli, making adipose tissue a relevant peripheral target tissue for obesity drugs (10, 37, 45) (99). In this report we investigated the potential of carotenoids to intensify the thermogenic response of human adipocytes to NR, a natural activator of PPARα and PPARγ. Pro-vitamin A carotenoids are converted into ligands for RXR nuclear receptors that form heterodimers with PPARs and coordinate expression of metabolism genes. We evaluated adipocytes treated for seven days with NR and carotenoids at concentrations that are reached in serum after oral administration to humans. The two pro-vitamin A carotenoids, BC and lutein, synergistically enhanced levels of UCP1 compared to NR alone. Lycopene, the third carotenoid that we investigated is not converted into an RXR ligand and did not alter the NR response. We used BC for additional experiments and found that NRBC also enhanced expression of ATGL, GLUT4, and adiponectin, which are drivers of lipolysis and insulin sensitivity. The effect of BC was selective. Expression of the PPARα target gene CPT1β, and PM20D1, a PPARγ regulated gene, were not elevated compared to treatment with NR alone.

NRBC upregulated protein levels of PGC-1α, PPARγ, and PPARα without comparable increases in mRNA levels, suggesting that the mechanism does not involve enhanced translation. Pgc-1α is a cold- induced coactivator for PPARγ and PPARα with a short protein half-life, and its transcriptional activity is upregulated in mice by mechanisms that slow degradation (58). When its protein levels increase, Pgc-1α protein associates with the PPARγ/RXR complex and selectively promotes expression of UCP1 and mitochondrial proteins (103). Elevated PGC-1α protein drives the PPARα/RXR complex towards GK expression and TG cycling activity in human adipocytes (104). The half-life of PPARγ and PPARα proteins is regulated by control of degradation rate after binding of ligands and cofactors (56, 57, 59). Since treatment with NR alone did not upregulate protein levels, our data suggest that NR and BC are both required to stabilize PPAR protein levels.

Brown and beige adipocytes release specialized bioactive lipokines and proteins into circulation that activate whole body insulin sensitivity and fatty acid uptake. Transcriptome sequencing showed that NRBC treatment substantially upregulated Cyp4f11, EPDX1 and EPDX2, enzymes that produce HETES and di-HOMEs (69, 72). Secreted HETES and di-HOMEs can signal tissues to increase uptake of fatty acids, and these actions have beneficial effects on lowering blood lipids (73). HETEs are strong PPAR activators (71), and when combined with the increased levels of PPAR proteins observed after NRBC treatment could amplify PPAR target gene expression. NRBC also induced ANGPTL4, a secreted protein that stimulates adipocyte lipolysis, and the insulin sensitizers adiponectin, FNDC4, and GDF11.

ATGL is the rate-limiting lipase for release of fatty acids from TGs, and fatty acids are natural ligands of PPARα and PPARу. In metabolically active beige cells, fatty acids are shuttled into mitochondria to fuel thermogenesis (87). In an energy-wasting futile cycle, fatty acids are also re-esterified onto the glycerol backbone of TGs. Whole transcriptome sequencing showed that NRBC stimulated a number of genes for glyceroneogenesis and TG synthesis, including PDK4, PCK1, GK, and GPD1. When thermogenesis is activated in adipocytes, PDK4 directs the flow of pyruvate generated by glycolysis into glycerol production (105). The PCK1 gene encodes PEPCK, the rate-limiting enzyme for glycerol synthesis from pyruvate. Studies using radio-labelled pyruvate in human adipocytes showed incorporation of the label into the glycerol backbone and into TGs after induction of PCK1 or PDK4 in human adipocytes (10, 106). In addition, GK and GPD1 encode enzymes that produce glycerol-3-phosphate, the substrate for fatty acid esterification (107, 108). The net result of TG cycling is elevated energy expenditure and a decrease of free fatty acids released into circulation (105).

In addition to UCP1 and TG cycling, we found that NRBC stimulated multiple other uncoupling and ATP-consuming enzymatic pathways. The most highly upregulated gene was PM20D1, encoding an enzyme that reversibly synthesizes NAAs that have potent mitochondrial uncoupling activity (109). In humans, the functional phenotype of PM20D1 is not well defined. In a clinical trial, serum concentrations of PM20D1 were positively correlated with adiposity and biomarkers of glucose metabolism such as glycated hemoglobin and fasting blood glucose (110). Both PM20D1 and NAAs are bound to serum proteins and regulation of circulating levels of NAAs in humans is complex (111). The relationship between adipose tissue PM20D1 and body weight in humans is unclear. Mutations in upstream PPARγ-binding regulatory elements of the PM20D1 gene cause a wide variation in basal expression levels in adipocytes. However, basal levels of PM20D1 in human adipose tissue do not correlate with circulating NAA levels (81). Presently, no clinical studies have been conducted to determine whether a substantial increase PM20D1 expression in thermogenic beige adipose tissue is sufficient to cause an increase in circulating NAA levels and contribute to weight loss.

The mitochondrial creatine kinases CKMT1A, CKMT1B and CKMT2 were highly induced by NRBC. Functional studies in mice showed that creatine kinases are upregulated in brown adipocytes after cold exposure or in the absence of Ucp1 and contribute to whole body energy expenditure (85). Interestingly, ablation of creatine metabolism in white adipose tissues inhibits thermogenesis and drives obesity in mice (112). In primary human brown adipocytes, proteomic analysis of thermogenesis pathways showed that ATP-coupled respiration is stimulated in parallel to uncoupled respiration and contributes half of the total oxygen consumed (86). Cycling of creatine phosphate supports ATP-coupled mitochondrial respiration by increasing the availability of ADP and phosphate to ATP-synthase. The strong induction of creatine kinases by NRBC suggests that ATP-coupled respiration and creatine phosphate cycling could contribute to thermogenesis in human beige adipocytes.

We showed that the enzymes involved in maintaining intracellular NAD levels, including AIFM2, NAMPT and NAPRT, increased after NRBC exposure. AIFM2 converts NADH to NAD to support the high levels required for glycolysis (89). NAMPT and NAPRT both synthesize NAD from intracellular precursors to support cellular needs during conditions of increased metabolic rate and oxidative stress (113). In addition, NAD is a cofactor for the sirtuin enzymes (SIRTs), which are protein deacetylases that regulate the activity of PPARγ, PGC-1α and other transcriptional activators of mitochondrial biogenesis and metabolism genes (114). Increasing NAD levels in human adipocytes can shift the phenotype from white to beige (95).

Our quantitative RNAseq analysis showed that the RXRα transcript is 300-fold more abundant than RXRγ in human white adipocytes, however, only the RXRγ isoform was robustly upregulated by NRBC treatment. We found that both RXRγ and RXRα proteins were bound in immunoprecipitated PPARγ complexes from untreated white adipocytes and in NRBC-treated beige cells. These results indicate that binding of both isoforms to the PPARγ complex is constitutive and ligand independent. In human perirenal adipose tissue, RXRγ expression is enriched in brown adipocyte progenitors and is induced in parallel with UCP1 during conversion of white to beige adipocytes (80). Possible mechanisms for targeting of beige genes could be recruitment of specific coactivators by RXRγ or conformational changes stimulated by BC binding that facilitate and stabilize interaction of this isoform with specific promoter elements (115) (46). In addition, the increase in RXRγ, PGC1α, PPARα and PPARγ proteins after NRBC treatment suggests the existence of a positive feedback loop that upregulates the genes identified in this study.

We used transcriptome sequencing data to estimate relative expression levels of receptors known to activate lipolysis or thermogenesis in adipocytes. In cells treated with NRBC, eight receptors were upregulated and several others trended higher. We evaluated stimulation of lipolysis with agonists for all receptors altered by NRBC and found that only a small subset were lipolytic. ACTH and adenosine stimulate lipolysis in murine white adipocytes and we saw no effect with either (44, 102). TGR5 and TRPM8 agonists induce thermogenesis genes in human adipocytes but did not stimulate lipolysis in our assay. We and others have shown that mild cold exposure of subcutaneous human adipocytes activates UCP1 expression by TRPM8, so the large increase in receptor levels after NRBC treatment has potential to act locally to sensitize adipocytes to cold and stimulate thermogenesis (45, 116). GHR and GPER1 were induced to the highest levels of all receptors after NRBC exposure, but neither growth hormone nor estrogen stimulated lipolysis.

NRBC upregulated PTHR levels. Moreover, PTH stimulated the greatest magnitude of lipolysis of all hormones tested in NRBC pretreated cells. PTH is released from parathyroid glands for regulation of systemic calcium homeostasis and all tissues express the PTHR (117). Evidence is growing that PTH plays an important role in adipose tissue metabolism. PTH stimulates lipolysis in mouse adipocytes and thermogenic gene expression in human adipocytes (99, 118). Cold-induced increases in circulating PTH shift whole-body metabolism toward lipid utilization to fuel energy expenditure in swimmers (119).

NRBC increased the stimulatory ratio of NPR1/NPR3 receptors. ANP is released after cold exposure and exercise and acts additively with adrenergic agonists to stimulate lipolysis and brown adipocyte characteristics in human white adipocytes (37). Although the increase was not statistically significant, our data showed a trend toward increased ANP-activated lipolysis in NRBC-treated cells.

We observed that the β1AR expression level in human adipocytes was substantially higher than the other βARs, and the β1AR was induced an additional three-fold by NRBC. We tested glycerol release with the pan-βAR agonist isoproterenol and the β1AR-selective agonist dobutamine and found that both stimulated a similar increase in NRBC-exposed cells compared to untreated. These results are in line with other studies showing that the β1AR is the dominant subtype in human white and brown adipocytes (39, 120).

NRBC significantly increased expression of PRKAR2B, the key PKA regulatory subunit linked to insulin sensitivity and resistance to weight gain in humans (88). We used 8-cpt-cAMP to activate PKA downstream of receptors and observed a considerable increase in the maximum capacity for lipolysis in adipocytes pretreated with NRBC. Impaired cAMP-stimulated TG lipolysis in subcutaneous adipose tissue is a characteristic of obesity and insulin resistance (121). A comprehensive analysis of two female cohorts with a ten-year follow-up was conducted to determine adipose tissue characteristics that predict weight gain. Low levels of stimulated lipolysis and PRKAR2B expression predicted weight gain and impaired glucose metabolism (122). Interestingly, there was no correlation with basal lipolysis or fat oxidation.

**Figure 9:**
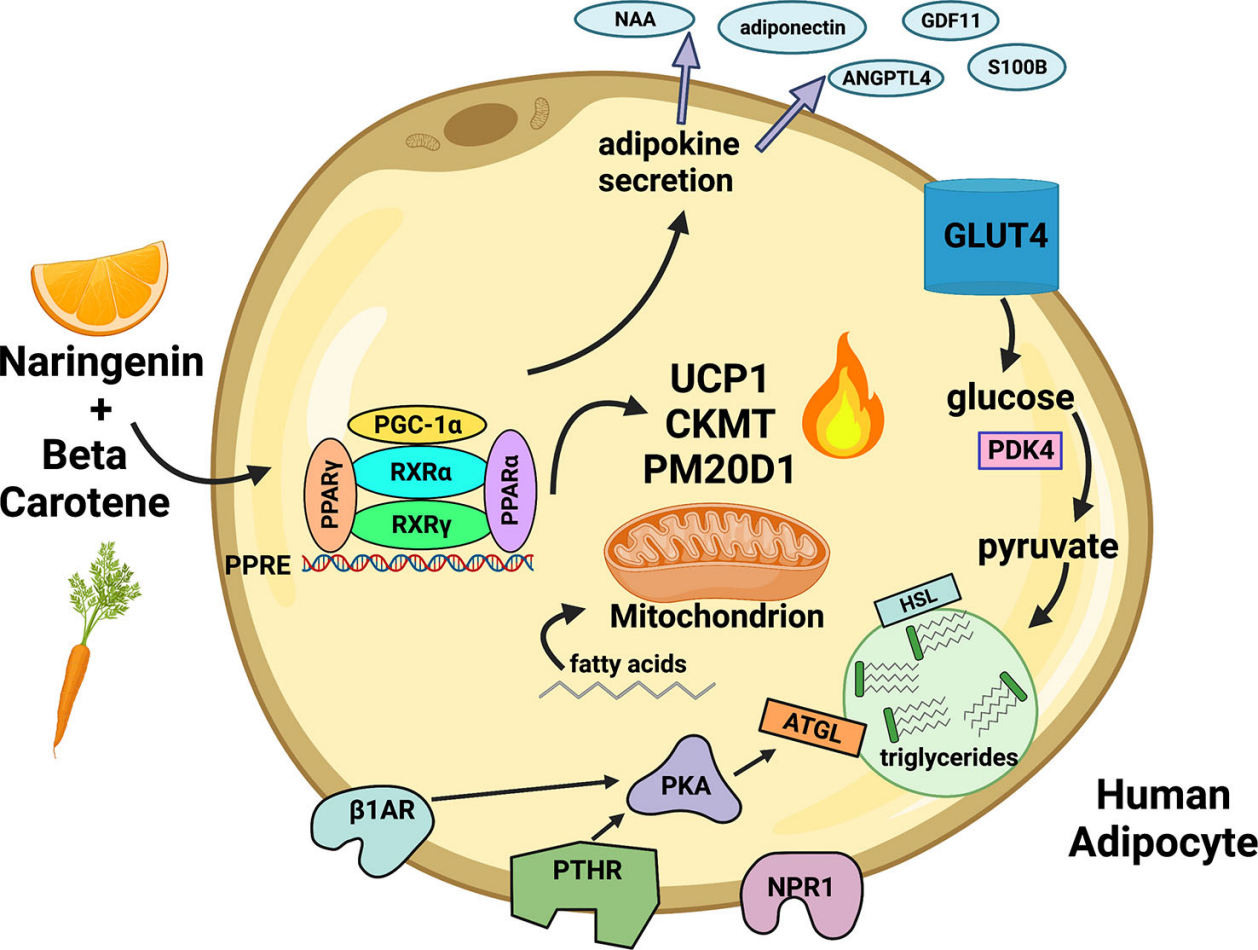
Proposed paradigm for remodeling of white adipocytes by NRBC. NR and BC bind nuclear receptors and activate gene expression at PPRE motifs. UCP1 and other uncoupling compounds and mitochondrial proteins mediate multiple energy-wasting enzymatic cycles that generate heat. PM20D1 regulates synthesis and degradation of N-acyl amino acids (NAA), molecules that directly uncouple mitochondria and increase energy expenditure. The synthesis and breakdown of creatine phosphate by the mitochondrial creatine kinases CKMT1A, CKMT1B and CKMT2 facilitates ATP-coupled respiration and enhances oxygen consumption. PDK4 directs pyruvate into synthesis of glycerol, fatty acids, and TGs to promote futile TG recycling. Lipolytic receptors and PKA are upregulated, increasing responsiveness to hormones and lipolysis. Fatty acids are transferred into mitochondria to fuel thermogenesis. Genes are turned on for production of bioactive peptides and lipokines which have autocrine insulin sensitizing effects and are secreted into circulation. NR (Naringenin), BC (β-carotene) Created with Biorender.com.

The human response to cold exposure and exercise involves transient release of ANP and PTH into circulation and sympathetic release of catecholamines in adipose tissues (100, 119) (37, 39, 99). Our data suggest that adipose tissue in an individual consuming NRBC would be more responsive to circulating hormones released after these stimuli. We demonstrate in this report that NRBC reprograms adipocytes by upregulating multiple thermogenic pathways, beneficial secreted factors, receptors and lipolysis, summarized in **Figure 9**. NR and BC have a good safety profiles and have the potential to be administered long-term without adverse effects (27, 29). A randomized, double blinded placebo-controlled clinical trial will be needed to determine whether the effects of NRBC on adipocytes will translate into weight loss and improvements in insulin sensitivity.

## Data availability statement

The datasets presented in this study can be found in online repositories. The names of the repository/repositories and accession number(s) can be found below: https://www.ncbi.nlm.nih.gov/geo/, GSE223313.

## Author contributions

Conceptualization, FG, AC and CR. Methodology: FG, AC, LC and CR. Formal analysis: AC, DZ, SG. Conducted research: AC, CC, SJ, CR, SG. Writing—original draft preparation: AC. Writing—review and editing: CR, YH, FG, CC. All authors contributed to the article and approved the submitted version.
