# Supplementary figures and images for: Naringenin and β-carotene convert human white adipocytes to a beige phenotype and elevate hormone- stimulated lipolysis
Source: Front Endocrinol (Lausanne). 2023 Apr 17;14:1148954. doi: 10.3389/fendo.2023.1148954 (PMC10153092; doi:10.3389/fendo.2023.1148954)

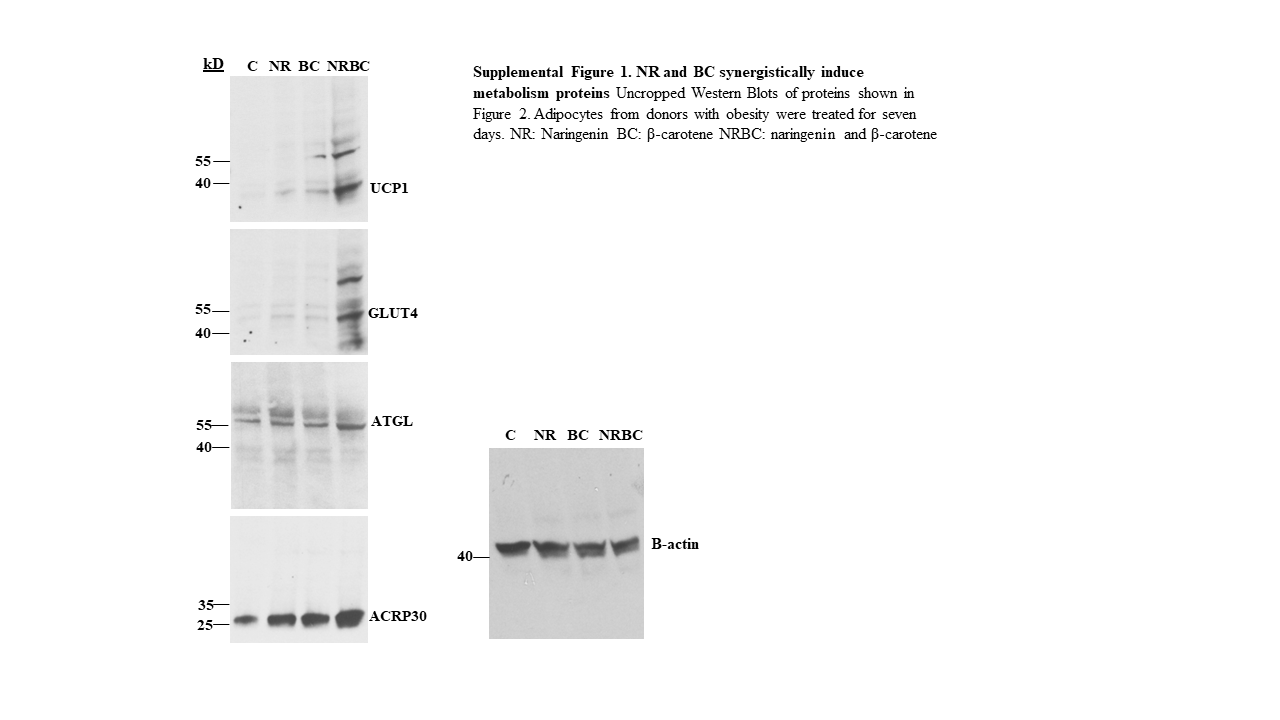

Supplement: Supplementary file 1 [file Image_1.tif]

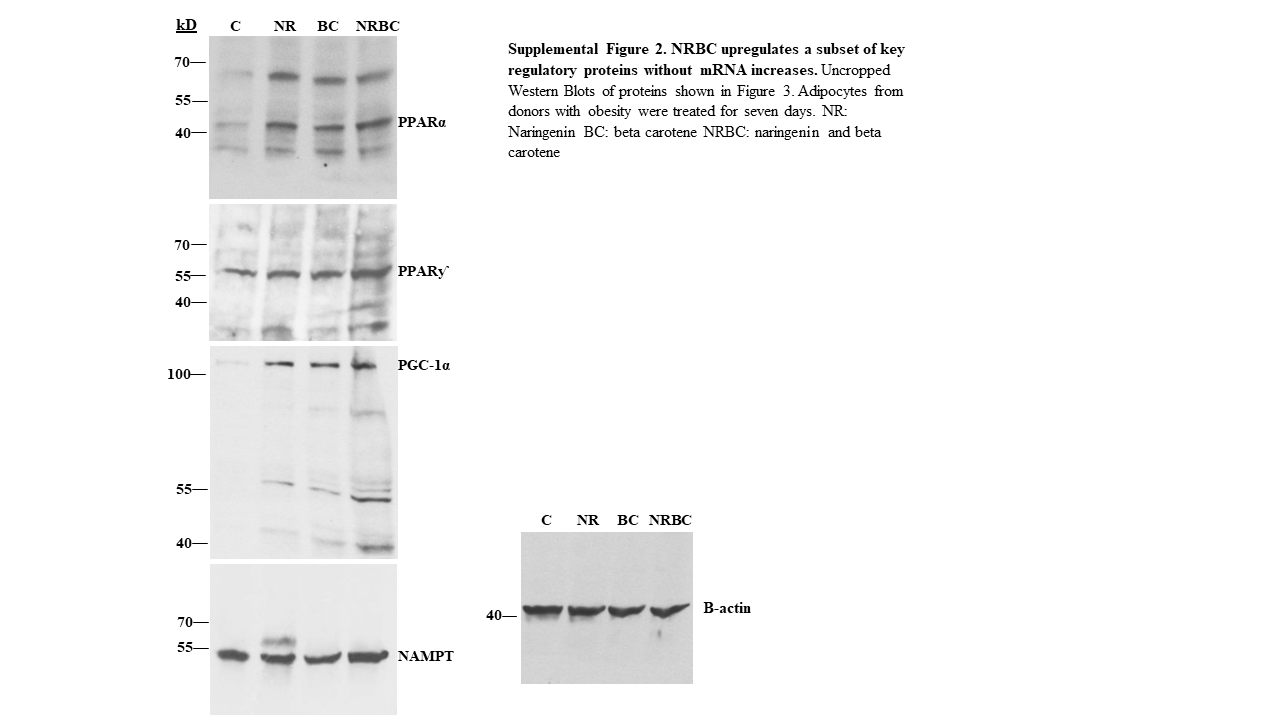

Supplement: Supplementary file 2 [file Image_2.tif]

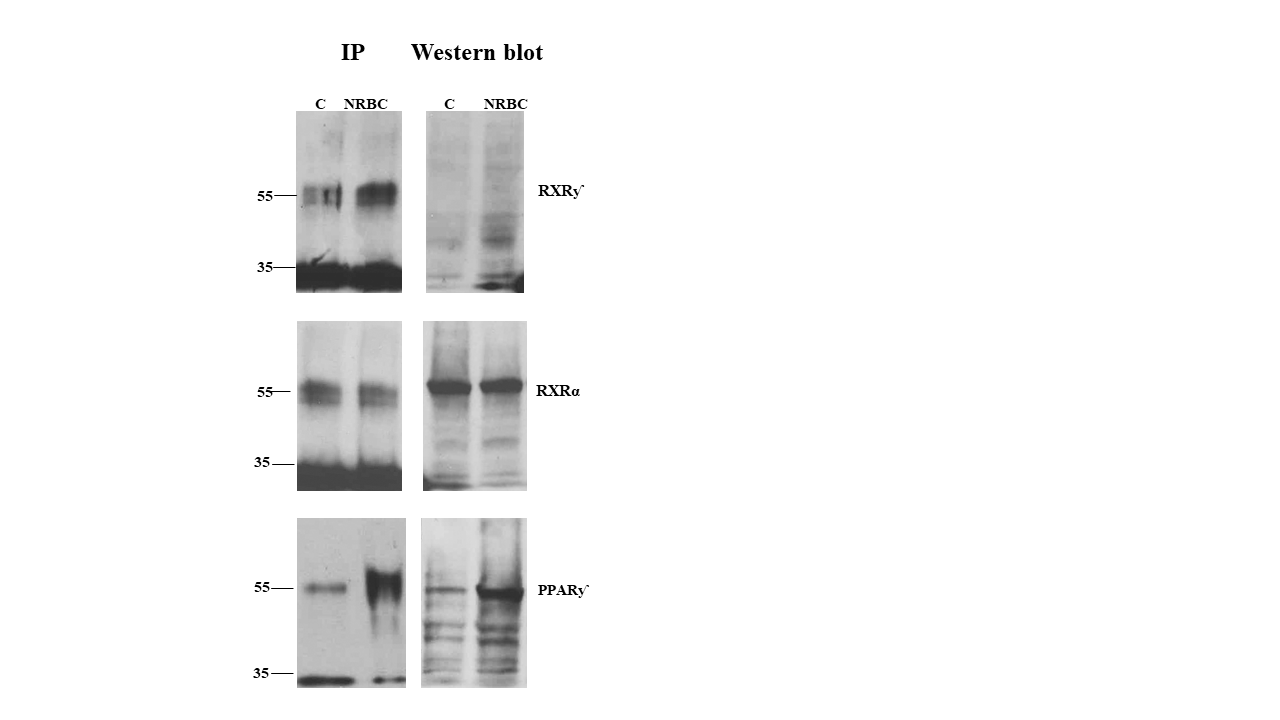

Supplement: Supplementary file 3 [file Image_3.tif]
